# Development of Blended Biopolymer-Based Photocatalytic Hydrogel Beads for Adsorption and Photodegradation of Dyes

**DOI:** 10.3390/gels9080630

**Published:** 2023-08-05

**Authors:** Seung Hyeon Weon, Jiwoo Han, Yong-Keun Choi, Saerom Park, Sang Hyun Lee

**Affiliations:** 1 Department of Biological Engineering, Konkuk University, Seoul 05029, Republic of Korea; shweon99@gmail.com (S.H.W.); jw991021@naver.com (J.H.); dragonrt@konkuk.ac.kr (Y.-K.C.); 2 R&D Center, ChoiLab Inc., Seoul 01811, Republic of Korea

**Keywords:** biopolymer, magnetic microbead, adsorption, photodegradation, photocatalyst

## Abstract

Blended biopolymer-based photocatalytic hydrogel beads were synthesized by dissolving the biopolymers in 1-ethyl-3-methylimidazolium acetate ([Emim][Ac]), adding TiO_2_, and reconstituting the beads with ethanol. The incorporation of modifying biopolymer significantly enhanced the adsorption capacity of the cellulose/TiO_2_ beads. Cellulose/carrageenan/TiO_2_ beads exhibited a 7.0-fold increase in adsorption capacity for methylene blue (MB). In contrast, cellulose/chitosan/TiO_2_ beads showed a 4.8-fold increase in adsorption capacity for methyl orange (MO) compared with cellulose/TiO_2_ beads. In addition, cellulose/TiO_2_ microbeads were prepared through the sol–gel transition of the [Emim][Ac]-in-oil emulsion to enhance photodegradation activity. These microbeads displayed a 4.6-fold higher adsorption capacity and 2.8-fold higher photodegradation activity for MB than the millimeter-sized beads. Furthermore, they exhibited superior dye removal efficiencies for various dyes such as Congo red, MO, MB, crystal violet, and rhodamine B, surpassing the performance of larger beads. To expand the industrial applicability of the microbeads, biopolymer/TiO_2_ magnetic microbeads were developed by incorporating Fe_2_O_3_. These magnetic microbeads outperformed millimeter-sized beads regarding the efficiency and time required for MB removal from aqueous solutions. Furthermore, the physicochemical properties of magnetic microbeads can be easily controlled by adjusting the type of biopolymer modifier, the TiO_2_ and magnetic particle content, and the ratio of each component based on the target molecule. Therefore, biopolymer-based photocatalytic magnetic microbeads have great potential not only in environmental fields but also in biomedical fields.

## 1. Introduction

Photocatalytic oxidation systems employing photocatalysts such as TiO_2_, ZnO, SnO_2_, and WO_3_ [1] have emerged as effective methods for degrading and mineralizing organic contaminants in wastewater. Under ultraviolet (UV) irradiation, these systems oxidize a wide range of organic and inorganic pollutants into harmless byproducts such as CO_2_ and H_2_O [2]. Among these photocatalysts, titanium dioxide (TiO_2_) is a semiconductor that occurs in three crystalline forms: anatase, rutile, and brookite [3]. When TiO_2_ is illuminated, it generates hydroxyl radicals on its surface, which possess strong oxidizing abilities and can degrade contaminants [3]. Due to its exceptional properties, including photoactivity, biological and chemical stability, non-toxicity, water insolubility, wide pH tolerance, and low cost, TiO_2_ has become one of the most extensively utilized and researched photocatalysts for the degradation of organic pollutants (e.g., phenols, dyes, herbicides, pesticides, and antibiotics) in aqueous environments [3,4,5,6]. However, heterogeneous photocatalytic processes involving TiO_2_ in sewage have the drawback of requiring a post-treatment step to recover the suspended photocatalysts, which can be laborious because of the nanoscale size of TiO_2_ particles. Therefore, it should be incorporated into support materials to achieve efficient photodegradation of pollutants using TiO_2_.

Biopolymer-based hydrogels, which are three-dimensional structures composed of hydrophilic biopolymer networks that retain large amounts of water, have attracted considerable attention as effective adsorbents. They are known for their high water retention capacity and low cost [7]. Recently, cellulose-based hydrogels have drawn particular interest in biomedical fields, such as drug delivery, tissue engineering scaffolds, biosensors, and adsorbents [8], owing to their excellent mechanical resistance and inherent eco-friendly properties, such as biodegradability and biocompatibility [9]. Furthermore, the cellulose hydrogel network containing −OH and −COOH groups can serve as an excellent support material, offering a robust platform for achieving highly dispersed TiO_2_ particles. However, the low solubility of cellulose in conventional organic solvents hinders the preparation of unmodified cellulose-based hydrogels by dissolving cellulose and regenerating it using antisolvents. In recent years, the development of new cellulose-dissolving solvents, such as ionic liquids (ILs) and deep eutectic solvents, such as alkylammonium hydroxide and alkylphosphonium hydroxide, has enabled the creation of various cellulose-based composite hydrogels [10].

Blending two or more biopolymers facilitates the facile preparation of biopolymer materials with novel physicochemical properties [11]. Furthermore, the properties of biopolymer materials can be controlled [12,13,14]. However, blending biopolymers through co-dissolution and regeneration poses challenges because of their low solubility in conventional solvents and different dissolution conditions. Recent advances in the ability of ILs to dissolve biopolymers have facilitated the preparation of blended biopolymer-based materials [15,16]. ILs exhibit high dissolving power for a wide range of biopolymers, including polysaccharides (e.g., cellulose, starch, dextran, agarose, and gums), proteins (e.g., gelatin, collagen, and silk), polynucleotides, and phenolic biopolymers (e.g., lignin), with or without co-solvents [15,16,17,18,19,20,21]. Wendler et al. and Liu et al. [12,13] reported the preparation of polysaccharide-blended materials using ILs that exhibit various characteristics depending on the biopolymer composition. Our group [14] obtained different biopolymer-blended hydrogel films by mixing cellulose as a support compound with polysaccharides, proteins, or phenolic biopolymers as a functional modifier. The resulting films exhibited distinct adsorption properties based on the type of biopolymer used. For example, the adsorption capacity of a cellulose/chitosan film for methyl orange was 9.1-fold higher, that of a cellulose/silk film for crystal violet was 7.9-fold higher, and that of a cellulose/chitosan film for lysozyme was 7.9-fold higher than that of cellulose film.

Recent advances in biomedicine have led to notable developments in the use of cellulose-based composite hydrogel photocatalysts. These photocatalysts have proven to be mild, efficient, and environmentally friendly, making them suitable for antibiotic degradation and sterilizing microorganisms [22]. Amaly et al. and Yue et al. [23,24] developed a cellulose composite aerogel doped with montmorillonite and a nanocellulose-based CdS/MoS_2_/montmorillonite composite for tetracycline degradation. Zhang et al. [25] reported on a cellulose/TiO_2_/β-CD composite hydrogel, demonstrating its effectiveness as a photo-activated antibacterial agent. These findings collectively highlight the promising potential of cellulose-based hydrogel photocatalysts for various biomedical applications. Cellulose-based TiO_2_ composite hydrogels in various forms have been prepared. Film forms of cellulose/carrageenan/TiO_2_ [26], cellulose/N-doped TiO_2_ [27], and cellulose/graphene oxide (GO)/TiO_2_ [28] composites were prepared to degrade methylene blue (MB). Wittmar et al. [29] fabricated cellulose/TiO_2_/Fe_3_O_4_ macrospheres using a mixture of [Emim][Ac] and dimethylsulfoxide (DMSO) to degrade rhodamine B. Monolithic forms of cellulose/TiO_2_ [30], cellulose/carboxymethyl cellulose/TiO_2_/Fe_3_O_4_ [31], and cellulose/Cu_2_O/TiO_2_/reduced graphene oxide (rGO) [32] composites were prepared and used for the degradation of MB and methyl orange (MO).

Micrometer-sized cellulose hydrogel beads have good potential for developing adsorbents, drug delivery carriers, chromatographic resins, and enzyme supports because of their large surface areas and hydrophilic properties [33]. Conventionally, cellulose hydrogel microspheres are prepared using a solvent-in-oil emulsion by the sol–gel transition method. Recently, we prepared cellulose hydrogel microspheres using [Emim][Ac] as a solvent for enzyme support [9]. The lipase immobilized on the cellulose hydrogel microspheres exhibited a significantly higher loading efficiency, immobilization yield, and specificity constant than that immobilized on microcrystalline cellulose or millimeter-sized hydrogel beads because of the larger surface area and favorable interactions of the cellulose microspheres. Additionally, we developed cellulose/biopolymer/Fe_3_O_4_ hydrogel microspheres to enhance enzyme adsorption capacity [34]. Liu et al. prepared cellulose/chitosan/Fe_3_O_4_ hydrogel microspheres for the immobilization of glucose oxidase [35], and Xue et al. investigated the immobilization of lysozyme on cellulose/Fe_3_O_4_ microspheres [36]. Thus, due to their favorable interactions with biomolecules, cellulose-based hydrogel microspheres exhibit significant potential as biomedical materials. However, incorporating TiO_2_ as a photocatalyst into cellulose-based microbeads remains unexplored.

**Figure 1:**
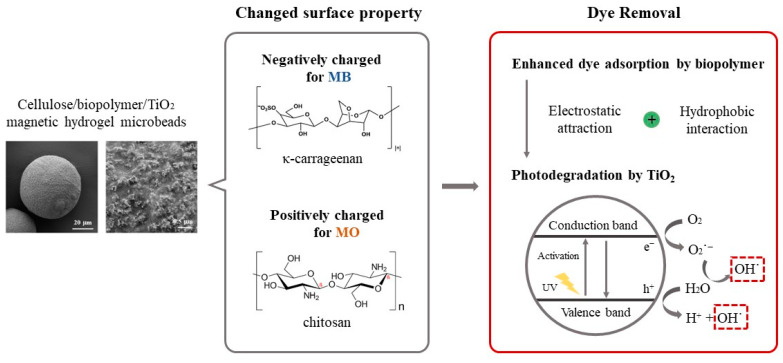
Schematic representation of the preparation of cellulose/biopolymer/TiO_2_ composite hydrogel beads and their dye removal mechanism.

In this study, we developed blended biopolymer-based photocatalytic hydrogel beads through the sol–gel transition of biopolymer solutions co-dissolved in [Emim][Ac]. We evaluated the adsorption capacity and photodegradation activity of the blended biopolymer/TiO_2_ hydrogel beads using various model dyes with different charges and hydrophobicities. Carrageenan, chitosan, and carbon nanotubes (CNT) were incorporated to modify the surface characteristics of the cellulose/TiO_2_ hydrogel beads. Furthermore, we prepared biopolymer/TiO_2_ magnetic hydrogel microbeads using an [Emim][Ac]-in-oil emulsion to enhance the efficiency of dye removal from aqueous solutions (Figure 1). The effects of the biopolymer charge, TiO_2_ content, magnetic particle content, and ratio of each component on the adsorption capacity and photodegradation activity of the composite hydrogel microbeads were investigated. We developed efficient and versatile photocatalytic hydrogel microbeads for environmental and biomedical applications by exploring the potential of blended biopolymers and incorporating TiO_2_ into hydrogel beads.

## 2. Results and Discussion

### 2.1. Preparation of Biopolymer/TiO_2_ Hydrogel Beads

In this study, biopolymer-based photocatalytic hydrogel beads were prepared via the co-dissolution of cellulose and a biopolymer (chitosan or carrageenan) using [Emim][Ac] as the biopolymer solvent and the dispersion of TiO_2_, followed by reconstitution with ethanol. Cellulose was used as the main supporting material for composite hydrogel beads because of its excellent mechanical strength and chemical stability. Chitosan and carrageenan were selected as modifiers to increase the positive and negative surface charges of the composite hydrogel beads, respectively. TiO_2_ nanoparticles were used as photocatalysts. The capacities of chitosan and carrageenan as charge modifiers were compared with those of CNT, which were used to increase the surface area of composite materials and enhance the photocatalytic activity of TiO_2_.

**Figure 2:**
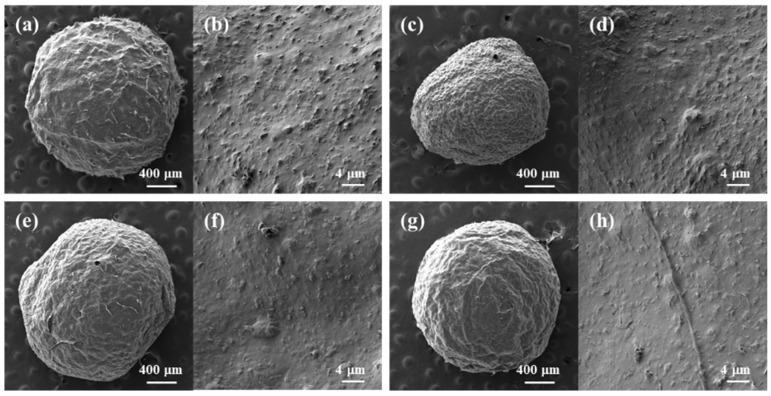
SEM images of freeze-dried biopolymer/TiO_2_ hydrogel beads. (**a**,**b**): cellulose/TiO_2_; (**c**,**d**): cellulose/CNT/TiO_2_; (**e**,**f**): cellulose/chitosan/TiO_2_; (**g**,**h**): cellulose/carrageenan/TiO_2_.

Millimeter-sized (ranging from 1.8 to 2.2 mm) composite hydrogel beads with the regular spherical shape of biopolymer/TiO_2_ were prepared using syringe extrusion and sol–gel transition by ethanol as an anti-solvent (Figure 2). Depending on the additives used, the surfaces of the prepared biopolymer/TiO_2_ composite beads exhibited no significant differences. All composite beads had nanosized bumpy surfaces due to the presence of TiO_2_. In contrast, cellulose, cellulose/chitosan and cellulose/carrageenan beads without TiO_2_ had smooth surfaces (Figure S1). The finely protruding surfaces of all the composite beads indicated that TiO_2_ was fully incorporated into the biopolymers in the composite beads during the sol–gel transition process. The X-ray diffraction (XRD) patterns of the cellulose beads, TiO_2_, and cellulose/TiO_2_ composite beads are shown in Figure S2. The XRD patterns revealed the crystal structure of TiO_2_ and confirmed its presence in the cellulose/TiO_2_ composite beads. The TiO_2_ nanoparticle can exist in three crystalline forms: anatase, rutile, and brookite. The main peak at 25.3° corresponded to the anatase form, which exhibited photocatalytic activity. These peaks were observed in the XRD patterns of cellulose/TiO_2_ composite beads. The XRD profiles of the cellulose and cellulose/TiO_2_ beads exhibited broad amorphous peaks, indicating a disruption in the crystalline nature of cellulose within the reconstituted beads. This disruption can be attributed to the breakage of internal hydrogen bonds in cellulose by [Emim][Ac].

### 2.2. Dye Removal of Biopolymer/TiO_2_ Hydrogel Beads through Adsorption and Photodegradation

The biopolymer/TiO_2_ hydrogel beads can remove various compounds from aqueous solutions via adsorption and photodegradation. To investigate the removal capacity of the target molecules of the biopolymer/TiO_2_, MB and MO were chosen as model dyes for positively and negatively charged molecules, respectively. The adsorption capacity (q_e_, mg/g) of the composite hydrogel beads was measured at 16 h, when the adsorption process reached equilibrium under dark conditions. The photocatalytic activity (k, min^−1^) of composite hydrogel beads was measured at an initial rate under ultraviolet (UV) irradiation after the adsorption process reached equilibrium under dark conditions. The dye removal efficiency (%) of the composite hydrogel beads was measured after 16 h of adsorption in the dark and after 6 h of UV irradiation.

Table 1 lists the MB adsorption capacity and photodegradation activity of the composite hydrogel beads. The adsorption capacity of the cellulose/carrageenan/TiO_2_ beads was 9.74 mg/g, which was 7.0 times higher than that of the cellulose/TiO_2_ beads. Carrageenan, with negatively charged sulfate groups (−OSO_3_^−^), induced electrostatic attraction with positively charged MB. In contrast, the adsorption capacity of the cellulose/chitosan/TiO_2_ beads was 8.2 times lower than that of the cellulose/TiO_2_ beads. Chitosan, with positively charged amine groups (−NH_3_^+^), exhibited electrostatic repulsion with MB [10,34]. These results indicated that blending biopolymers with cellulose using [Emim][Ac] efficiently changed the surface charge of the cellulose/TiO_2_ hydrogel beads without chemical modification. Traditional chemical modification methods can alter the biocompatibility and biodegradability of cellulose. The adsorption capacity of the cellulose/CNT/TiO_2_ beads was 4.9-fold higher than that of the cellulose/TiO_2_ beads. The enhanced adsorption capacity of the composite beads containing CNT can be explained by their large surface area and the hydrophobicity of CNT [37]. The highest adsorption capacity was obtained with cellulose/carrageenan/TiO_2_.

The photodegradation activity of the cellulose/TiO_2_ beads was similar to that of cellulose/CNT/TiO_2_. In contrast, cellulose/carrageenan/TiO_2_ beads showed the lowest photocatalytic activity. The lowest k value of the cellulose/carrageenan/TiO_2_ beads may be attributed to the inhibition of UV light transmission into the TiO_2_ particles on the internal side of the beads due to the large number of MB molecules adsorbed on the surface of the composite beads. The inhibition of UV light transmission can also occur in cellulose/CNT/TiO_2_ beads, which exhibit a high MB adsorption capacity. However, the enhanced photocatalytic activity of TiO_2_ in the presence of CNT has been reported [38], which may compensate for the decreased photocatalytic activity caused by the inhibition of UV transmission. The photocatalytic activity of the cellulose/chitosan/TiO_2_ beads was similar to that of the cellulose/TiO_2_ beads. However, their MB adsorption capacities were deficient. These results indicate that the blended modifier did not significantly influence the photocatalytic activity of the composite beads for MB; however, a large amount of MB adsorbed on the composite surface could decrease the k value. The highest dye removal efficiency of 67.4% was obtained with the cellulose/carrageenan/TiO_2_ beads, which showed a significantly enhanced MB adsorption capacity. In contrast, the dye removal efficiency of cellulose/chitosan/TiO_2,_ which showed the lowest MB adsorption capacity, was only 19.6%. The dye-removal efficiency of the cellulose/CNT/TiO_2_ beads was similar to that of the cellulose/TiO_2_ beads.

Table 2 lists the MO adsorption capacity and photodegradation activity of the composite hydrogel beads. The adsorption capacity of cellulose/chitosan/TiO_2_ beads was 0.96 mg/g, which was 4.8 times higher than that of cellulose/TiO_2_ beads, whereas that of cellulose/carrageenan/TiO_2_ beads was 2.0 times lower than that of cellulose/TiO_2_ beads. These results can be explained by the attraction between positively charged chitosan and negatively charged MO and the repulsion between negatively charged carrageenan and MO. Therefore, chitosan and carrageenan can be successfully used as positive and negative surface modifiers, respectively, for the cellulose/TiO_2_ composite beads. The cellulose/CNT/TiO_2_ beads showed the highest adsorption capacity for MO, and their q_e_ value was 8.2-fold higher than that of the cellulose/TiO_2_ beads. The enhanced adsorption capacity of the composite beads containing CNT may be explained by the large surface area of CNT and the hydrophobic interactions of CNT with MO, which was more hydrophobic than MB.

The MO photodegradation activity of the composite hydrogel beads decreased as their adsorption capacity increased. The cellulose/CNT/TiO_2_ beads, which exhibited the highest MO adsorption capacity, exhibited the lowest photodegradation activity. The cellulose/carrageenan/TiO_2_ beads showed photocatalytic activity similar to that of cellulose/TiO_2_. However, they showed the lowest adsorption capacity for MO. These results can be explained by the limitations of UV light transmission due to the adsorption of MO molecules on the surface of the composite beads. The cellulose/chitosan/TiO_2_ beads exhibited the highest dye removal efficiency (58%). Although the cellulose/chitosan/TiO_2_ beads demonstrated lower photodegradation activity than the cellulose/TiO_2_ beads, their higher adsorption capacity could explain this discrepancy. In contrast, the cellulose/CNT/TiO_2_ beads, despite having the highest adsorption capacity, displayed the lowest dye removal efficiency (39%). This can be attributed to their low photodegradation activity.

Cellulose/TiO_2_ composite hydrogel beads exhibited enhanced MB and MO dye removal efficiencies when blended with carrageenan and chitosan, respectively. However, their enhanced dye removal efficiency could be mostly caused by the increased adsorption capacity, because their photodegradation activity was lower than that of the cellulose/TiO_2_ beads. When dye molecules are adsorbed in large quantities on the surfaces of the composite beads, they can impede the transmission of UV light into the TiO_2_ particles present within the beads. This problem can be addressed by using smaller-sized biopolymer/TiO_2_ beads, which can significantly enhance the efficiency of dye removal.

### 2.3. Preparation and Dye Removal of Cellulose/TiO_2_ Hydrogel Microbeads

To overcome the inhibition of light transmission caused by high dye concentrations in millimeter-sized cellulose/TiO_2_ beads, we prepared micrometer-sized cellulose/TiO_2_ beads through a sol–gel transition, using an [Emim][Ac]-in-oil emulsion. The adsorption capacity of the cellulose/TiO_2_ microbeads was 6.41 mg/g (Table 3), whereas the q_e_ value of the millimeter-sized cellulose/TiO_2_ beads was 1.40 mg/g under the same adsorption conditions (Table 1). Microbeads showed 4.6-fold higher adsorption capacity than millimeter-sized beads. In addition, the adsorption of the microbeads reached equilibrium in 1 h, whereas that of the millimeter beads required 16 h to reach equilibrium. The photodegradation activity of the cellulose/TiO_2_ microbeads was 2.8-fold higher than that of the millimeter-sized cellulose/TiO_2_ beads (Table 1 and Table 3). Microbeads showed much higher photodegradation activity than millimeter-sized beads, although the amount of MB adsorbed on the microbeads was much higher than that adsorbed on the millimeter-sized beads. These results clearly indicated that the size reduction in the cellulose/TiO_2_ hydrogel beads could overcome the inhibition of light transmission caused by MB adsorbed in large quantities on the surfaces of the composite beads. The dye removal efficiency of the cellulose/TiO_2_ microbeads was 95%, which was much higher (40%) than that of millimeter-sized cellulose/TiO_2_ beads (Table 1 and Table 3).

**Figure 3:**
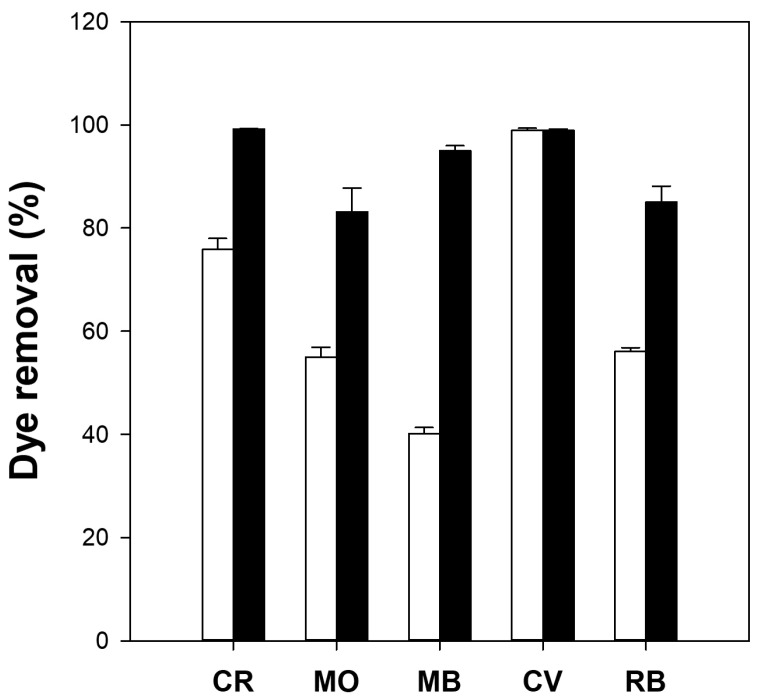
Dye removal efficiency (%) of millimeter-size (white bars) and micrometer-size (black bars) cellulose/TiO_2_ hydrogel beads. Reaction conditions: 5 mg beads, 10 mg/L dye (Congo red [CR], methyl orange [MO], methylene blue [MB], crystal violet [CV] and rhodamine B [RB]) solution, and 16 h of adsorption in the dark, followed by 6 h of UV irradiation.

The dye removal capacity of the cellulose/TiO_2_ microbeads was measured using various dyes and compared with that of millimeter-scale cellulose/TiO_2_ beads. The adsorption capacities (q_e_) of the millimeter-sized cellulose/TiO_2_ beads for Congo red (CR), MO, MB, crystal violet (CV), and rhodamine B (RB) were 3.5, 0.2, 0.6, 2.1, and 0.3 mg/g, respectively. Conversely, cellulose/TiO_2_ microbeads exhibited q_e_ values of 6.8, 0.3, 3.0, 2.8, and 1.2 mg/g for CR, MO, MB, CV, and RB, respectively. The adsorption capacity of the microbeads was significantly higher, ranging from 1.3 to 5.0 times greater than that of the millimeter-sized beads. The dye removal efficiency of the cellulose/TiO_2_ microbeads was much higher than that of the millimeter-sized cellulose/TiO_2_ beads for all tested dyes (Figure 3). For example, microbeads showed 95% dye removal for MB, whereas millimeter-sized beads showed only 40% dye removal. These results indicated that cellulose/TiO_2_ microbeads with a larger specific surface area than millimeter-sized beads could efficiently remove some dyes from aqueous solutions through higher adsorption capacity and photodegradation activity. This phenomenon applies to other chemicals and biomolecules. Kim et al. [39] and Jo et al. [9] reported that small-sized cellulose-based materials can enhance protein adsorption compared with larger-sized materials because of their improved specific surface area. Although cellulose/TiO_2_ microbeads exhibit superior adsorption capacity and photodegradation activity compared with millimeter-sized cellulose/TiO_2_ beads, because of their small size, industrial applications of microbeads may be constrained by the challenges associated with their recovery and reusability. The development of magnetic microbeads that can be easily separated using a magnet has the potential to overcome the limitations of industrial applications of microbeads.

### 2.4. Preparation and Dye Removal of Cellulose/TiO_2_ Magnetic Hydrogel Microbeads

To improve the recovery and reusability of the microbeads, we prepared cellulose/TiO_2_/Fe_2_O_3_ microbeads by dispersing both TiO_2_ and iron (III) oxide in an [Emim][Ac]-in-oil emulsion. The cellulose/TiO_2_ magnetic hydrogel microbeads, containing more than 0.5% Fe_2_O_3_ in the bead-forming solution, were easily collected within 10 min using a magnet (Figure S3).

**Figure 4:**
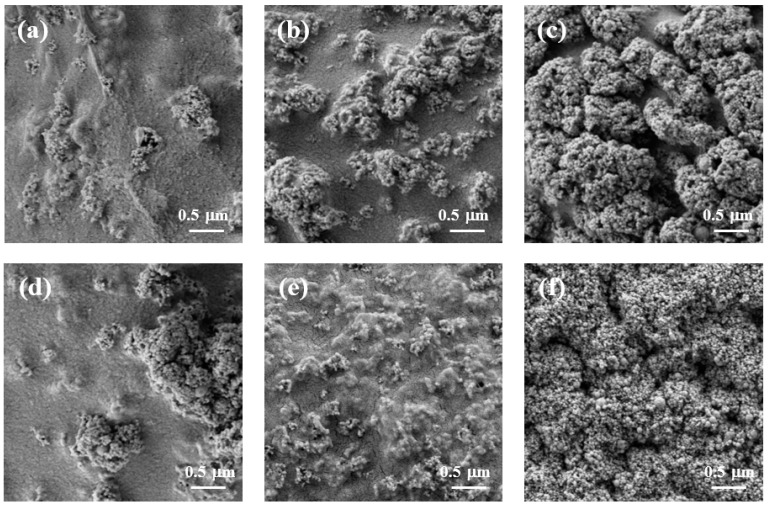
SEM images of freeze-dried cellulose/TiO_2_ magnetic microbeads. (**a**) 0.5% TiO_2_; (**b**) 0.5% TiO_2_ and 0.1% Fe_2_O_3_; (**c**) 0.5% TiO_2_ and 1% Fe_2_O_3_; (**d**) 0.5% TiO_2_ and 0.5% Fe_2_O_3_; (**e**) 3.5% TiO_2_ and 0.5% Fe_2_O_3_; (**f**) 7% TiO_2_ and 0.5% Fe_2_O_3_.

Figure 4 shows the surface morphology of cellulose/TiO_2_ magnetic microbeads with the increasing content of Fe_2_O_3_. The surfaces of the cellulose/TiO_2_ magnetic microbeads appeared rough, with clustered and aggregated formations scattered across various locations. This tendency became more pronounced as the amount of Fe_2_O_3_ increased at a fixed TiO_2_ content (0.5%) (Figure 4a–c). The increase in the amount of used Fe_2_O_3_ and the consequent increase in the amount of exposed Fe_2_O_3_ on the surface led to a decrease in the photodegradation activity of TiO_2_ by inhibiting light transmission. However, Fe_2_O_3_ exposed on the surface of cellulose/TiO_2_ magnetic microbeads can act as both an adsorbent and a photocatalyst [40].

Table 3 shows the effect of the magnetic particle content of the cellulose/TiO_2_ magnetic microbeads on the adsorption capacity and photodegradation activity of MB. Both adsorption capacity and photocatalytic activity were significantly influenced by the magnetic particle content. Adding Fe_2_O_3_ up to 0.5% decreased the adsorption capacity. In contrast, microbeads prepared with more than 0.5% showed higher q_e_ values than those prepared with 0.5% Fe_2_O_3_. The q_e_ value of the microbeads prepared with 1% Fe_2_O_3_ was 1.2 times higher than that of cellulose/TiO_2_ microbeads without Fe_2_O_3_. The surfaces of the cellulose/TiO_2_ magnetic microbeads prepared using 1% Fe_2_O_3_ were covered with aggregated Fe_2_O_3_ (Figure 4). The Fe_2_O_3_ aggregates exposed on the surface of the magnetic microbeads may have increased the adsorption capacity for MB because Fe_2_O_3_ has been reported as a good adsorbent for MB [40]. The photodegradation activity of the cellulose/TiO_2_ magnetic microbeads decreased with increasing the content of Fe_2_O_3_. The k value of the cellulose/TiO_2_ microbeads prepared with only 0.1% Fe_2_O_3_ was 2.5-fold lower than that of the cellulose/TiO_2_ microbeads prepared without Fe_2_O_3_. Although the photocatalytic activity of F_2_O_3_ for MB has been reported [40], adding Fe_2_O_3_ decreased the photodegradation activity of cellulose/TiO_2_ magnetic microbeads. Including Fe_2_O_3_ in the cellulose/TiO_2_ magnetic microbeads did not significantly improve their adsorption capacity or photodegradation activity. Consequently, the subsequent experiments used the minimum amount of Fe_2_O_3_ required to serve as magnetic particles. Adding a minimum of 0.5% Fe_2_O_3_ to the gel-forming solution enabled rapid separation using a magnet.

To investigate the effect of the TiO_2_ content of the cellulose/TiO_2_ magnetic microbeads on MB adsorption capacity and photodegradation activity, the TiO_2_ content was varied from 0.5% to 7% at a fixed content of Fe_2_O_3_ (0.5%). The surface morphology and mean diameter of the prepared cellulose/TiO_2_ magnetic microbeads changed significantly depending on the content of TiO_2_. The surfaces of the cellulose/TiO_2_ magnetic microbeads had a rough texture with scattered aggregated particles. This tendency became more pronounced as the amount of TiO_2_ increased at a fixed Fe_2_O_3_ content (0.5%) (Figure 4d–f). The surfaces of cellulose/TiO_2_ magnetic microbeads, prepared with 7% TiO_2_, were covered entirely with aggregated TiO_2_ nanoparticles. As the amount of TiO_2_ increased, the amount of exposed TiO_2_ on the surface increased, which could have led to an increase in the photodegradation activity of TiO_2_. However, the adsorption capacity of the cellulose/TiO_2_ magnetic microbeads for MB could be decreased by decreasing the interactions between the cellulose and dye molecules because of the decreased cellulose content on the surface. The mean diameter of the cellulose/TiO_2_ magnetic microbeads increased proportionally with TiO_2_ content. The microbead diameters ranged from 22.3 mm to 52.6 mm, depending on the TiO_2_ content, which varied from 0.5% to 7.0%. A strong correlation exists between the mean diameter of the microbeads and the TiO_2_ content (r^2^ = 0.960, *n* = 5).

Table 4 shows the effect of TiO_2_ content in the cellulose/TiO_2_ magnetic microbeads on the adsorption capacity and photodegradation activity of MB. When the TiO_2_ content was 3.5%, the adsorption capacity and photodegradation activity were the highest. The q_e_ and k values increased as the TiO_2_ content increased from 0.5% to 3.5%. However, beyond 3.5% TiO_2_ content, both values decreased with a further increase in TiO_2_ content up to 7%. As TiO_2_ content increased, the mean diameter of the microbeads increased, whereas their specific surface area decreased. In addition, a noticeable transformation of the surface morphology was observed when the TiO_2_ content exceeded 3.5%. Larger bead size and altered surface morphology may impede the interactions between cellulose and MB and hinder light transmission. To assess the efficiency of TiO_2_ as a photocatalyst in the cellulose/TiO_2_ magnetic microbeads, the specific rate constant was calculated based on the same TiO_2_ content (k/mg TiO_2_). Microbeads prepared with 3.5% TiO_2_ exhibited the highest photodegradation activity, whereas those prepared with 0.5% TiO_2_ showed the highest specific rate constant. The specific rate constant of the microbeads prepared with 3.5% TiO_2_ was 3.2 times lower than that of those prepared with 0.5% TiO_2_. These results could be attributed to several factors, including increased bead size, alterations in surface morphology, and TiO_2_ aggregation. The increased bead size resulting from the higher TiO_2_ content led to a decrease in the specific surface area of the microbeads. Furthermore, as the TiO_2_ content increased, a significant amount of TiO_2_ aggregated on the microbead surfaces. These aggregated forms of TiO_2_ may have diminished the photocatalytic activity due to overlapping, thereby reducing overall efficiency. Wittmar and Ulbricht [29] reported the limitation of increasing the TiO_2_ content in cellulose films due to the agglomeration of TiO_2_.

### 2.5. Preparation and Dye Removal of Biopolymer/TiO_2_ Magnetic Hydrogel Microbeads

**Figure 5:**
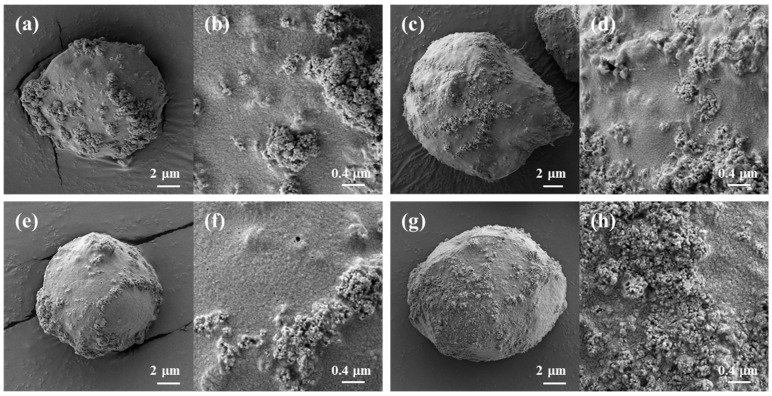
SEM images of freeze-dried biopolymer/TiO_2_ magnetic microbeads. (**a**,**b**): cellulose/TiO_2_/Fe_2_O_3_; (**c**,**d**): cellulose/CNT/TiO_2_/Fe_2_O_3_; (**e**,**f**): cellulose/chitosan/TiO_2_/Fe_2_O_3_; (**g**,**h**): cellulose/carrageenan/TiO_2_/Fe_2_O_3_.

The preparation conditions for the cellulose/TiO_2_ magnetic microbeads were optimized for MB removal. However, the adsorption capacity and photodegradation activity of cellulose/TiO_2_ magnetic microbeads for other molecules could be improved by blending specific biopolymers as modifiers for the target molecules. Chitosan, CNT, and carrageenan were added to cellulose/TiO_2_ magnetic microbeads to improve the dye removal efficiency of MB and MO. Figure 5 shows the surfaces of freeze-dried biopolymer/TiO_2_ magnetic microbeads. The surfaces of all the microbeads exhibited roughness, with scattered aggregated formations occurring in various areas due to the presence of exposed TiO_2_ and Fe_2_O_3_. However, no significant differences were observed among the biopolymer/TiO_2_ magnetic microbeads. The surface of the magnetic microbeads was rougher than that of the millimeter-sized beads (Figure 2), which could be attributed to the presence of exposed TiO_2_ and Fe_2_O_3_ on the surface of the magnetic microbeads.

Table 5 shows the MB adsorption and photodegradation capacities of the biopolymer/TiO_2_ magnetic microbeads for MB. The adsorption capacity of the negatively charged cellulose/carrageenan microbeads was 5.8 times higher than that of cellulose microbeads. In contrast, the adsorption capacity of the cellulose-chitosan microbeads was 1.1 times lower than that of the cellulose microbeads. Although the repulsion between the positively charged chitosan and MB significantly decreased the adsorption capacity for MB in millimeter-sized beads (Table 1), the TiO_2_ and Fe_2_O_3_ exposed on the surface of the cellulose/chitosan magnetic microbeads may have increased the adsorption capacity for MB. The cellulose/CNT/TiO_2_ magnetic microbeads exhibited the highest adsorption capacity, due to the large surface area and hydrophobicity of the CNT. Adding CNT, chitosan, and carrageenan to the cellulose/TiO_2_ magnetic microbeads decreased the photodegradation activity. The lower photodegradation activities of the cellulose/CNT and cellulose/carrageenan magnetic microbeads may have been caused by the inhibition of UV light transmission by the MB molecules adsorbed in large quantities on the surface of the microbeads. Although microbeads partly overcome the inhibition of UV transmission, the much higher adsorption rate of microbeads compared with millimeter-sized beads may limit the photodegradation activity of magnetic microbeads. The cellulose/carrageenan magnetic microbeads showed the highest dye removal efficiency of 74.8%. In contrast, cellulose/chitosan showed the lowest dye removal efficiency (32.0%). The dye removal efficiency of the cellulose/CNT magnetic microbeads was 65.4%, which was 1.4 times higher than that of the cellulose magnetic microbeads. This may have been caused by the 5.8-fold enhanced adsorption capacity of the cellulose/CNT microbeads compared with that of the cellulose microbeads.

The MB removal capacities of the biopolymer/TiO_2_ magnetic microbeads (Table 5) were compared with those of millimeter-sized biopolymer/TiO_2_ beads (Table 1) under the same reaction conditions, except for the adsorption time. The magnetic microbeads and millimeter-sized beads required 1 h and 16 h to reach equilibrium, respectively. Except for cellulose/carrageenan, the adsorption capacities of the magnetic microbeads were higher than those of the millimeter-sized beads. The adsorption capacity of the cellulose/carrageenan microbeads was not significantly different from that of the millimeter-sized beads. The presence of TiO_2_ and Fe_2_O_3_ on the surface of the cellulose/carrageenan microbeads could have impeded the interactions between carrageenan and MB. These results indicated that magnetic microbeads could adsorb MB more efficiently and in a shorter time than millimeter-sized beads. The photodegradation activities of the microbeads, except for the cellulose beads, were lower than those of the millimeter-sized beads. The cellulose microbeads showed a 1.2-fold higher k value than that of the millimeter-sized cellulose beads. The lower photodegradation activity of the microbeads, except for the cellulose microbeads, may have been caused by the presence of Fe_2_O_3_. Although it has been reported that Fe_2_O_3_ can act as a photocatalyst [40], the addition of Fe_2_O_3_ to cellulose/TiO_2_ microbeads decreased the photodegradation activity (Table 3). The dye removal efficiencies of all microbeads were higher than those of the millimeter-sized beads. These results indicated that the magnetic microbeads removed MB from the aqueous solution more efficiently and in a shorter time than the millimeter-sized beads.

Table 6 shows the adsorption capacity and photodegradation activity of biopolymer/TiO_2_ magnetic microbeads for MO. The adsorption capacity of the positively charged cellulose/chitosan microbeads was 4.5 times higher than that of the cellulose microbeads. In contrast, the adsorption capacity of the cellulose/carrageenan microbeads was 5.7 times lower than that of the cellulose microbeads. The cellulose/CNT/TiO_2_ magnetic microbeads exhibited the highest adsorption capacity, owing to the large surface area and hydrophobicity of the CNT. The addition of chitosan and carrageenan to the cellulose/TiO_2_ magnetic microbeads increased their photodegradation activity. The decreased photodegradation activity of the cellulose/CNT magnetic microbeads may have been caused by the inhibition of UV light transmission by MO molecules adsorbed in large quantities on the surface of the microbeads. The cellulose/chitosan magnetic microbeads had the highest dye removal efficiency (57.3%). In contrast, the cellulose/CNT magnetic microbeads showed the lowest dye removal (30.2%).

The MO removal capacities of the biopolymer/TiO_2_ magnetic microbeads (Table 6) were compared with those of the millimeter-sized biopolymer/TiO_2_ beads (Table 2) under the same reaction conditions, except for the adsorption time. The magnetic microbeads and millimeter-sized beads required 1 h and 16 h to reach equilibrium, respectively. The adsorption capacities of the magnetic microbeads, except for the cellulose/carrageenan beads, were higher than those of the millimeter-sized beads. The cellulose/carrageenan magnetic microbeads exhibited an adsorption capacity similar to that of millimeter-sized beads. The photodegradation activity of the cellulose/chitosan microbeads was 1.1-fold higher than that of the millimeter-sized beads. The k values of the cellulose and cellulose/carrageenan microbeads were lower than those of the millimeter-sized beads. The dye removal efficiencies of the magnetic microbeads, except for the cellulose microbeads, were similar to those of the millimeter-sized beads. Although the biopolymer/TiO_2_ magnetic microbeads showed a much higher dye removal efficiency for MB than the millimeter-sized beads, their dye removal efficiency for MO was similar to or lower than that of the millimeter-sized beads. This could have been due to the presence of Fe_2_O_3_ because cellulose/TiO_2_ microbeads without Fe_2_O_3_ showed a much higher dye removal efficiency for MO than millimeter-sized beads (Figure 3). The morphological differences between the microbeads and millimeter-sized beads changed their adsorption capacity and photodegradation activity. In addition, the surfaces of the microbeads were covered with more TiO_2_ and Fe_2_O_3_ than those of the millimeter-sized beads (Figure 2 and Figure 5). The surface of the microbeads, prepared through the sol–gel transition of the [Emim][Ac]-in-oil emulsion, can be changed using biopolymers as additives because the charge of the added biopolymers can influence the formation of the emulsion.

Table 7 compares the recently reported dye removal efficiencies achieved by cellulose/TiO_2_-based photocatalytic hydrogels. Most studies have been conducted using hydrogels in the form of films or monoliths; studies on photocatalytic hydrogels using cellulose microbeads or blended biopolymers are scarce. A direct comparison is not feasible because of variations in the dye concentrations and hydrogel compositions used in each study. However, the blended biopolymer-based photocatalytic microbeads developed in this study exhibited comparable dye removal efficiencies to other forms of hydrogels. Microbead-shaped photocatalytic hydrogels have potential advantages for applications in the biomedical field, such as protein support and photo-antibacterial agents, thus expanding the scope of photocatalytic hydrogel applications.

## 3. Conclusions

In this study, we successfully synthesized blended biopolymer-based photocatalytic hydrogel beads by co-dissolving biopolymers in [Emim][Ac], followed by the dispersion of TiO_2_ and reconstitution with ethanol. Incorporating carrageenan and chitosan into the cellulose/TiO_2_ hydrogel beads significantly enhanced their MB and MO adsorption capacities. However, many dye molecules adsorbed on the surface of the millimeter-sized composite beads hindered UV light transmission, resulting in reduced photodegradation activity.

To overcome this limitation, we developed cellulose/TiO_2_ hydrogel microbeads through the sol–gel transition of an [Emim][Ac]-in-oil emulsion. These microbeads exhibited 4.6- and 2.8-fold higher adsorption capacities and photodegradation activities, respectively, than millimeter-sized beads. Furthermore, the cellulose/TiO_2_ microbeads demonstrated superior dye removal efficiency for a wide range of dyes, including CR, MO, MB, CV, and RB, surpassing the performance of millimeter-sized beads. This demonstrated that the reduction in the size of the cellulose/TiO_2_ hydrogel beads successfully overcame the limitation of light transmission, leading to improved photocatalytic performance.

Moreover, we expanded the industrial applicability of the cellulose/TiO_2_ microbeads by incorporating their magnetic properties. By optimizing the content and ratio of TiO_2_ and Fe_2_O_3_, we could fine-tune the adsorption capacity and photodegradation activity of the cellulose/TiO_2_ magnetic microbeads. In addition, we explored the potential of blended, biopolymer-based, photocatalytic magnetic microbeads to further enhance the dye removal efficiency of cellulose/TiO_2_ magnetic microbeads. The cellulose/carrageenan/TiO_2_ magnetic microbeads exhibited significantly higher dye removal efficiencies than the cellulose/TiO_2_ magnetic microbeads for MB.

The microbeads outperformed the millimeter-sized beads regarding the efficiency and time required for dye removal from aqueous solutions. Furthermore, the physicochemical properties of the biopolymer-based magnetic microbeads can be easily controlled by altering the type of modifier, adjusting the TiO_2_ and magnetic particle contents, and varying the ratio of each component based on the target molecule. These findings highlight the potential of biopolymer-based photocatalytic magnetic microbeads for diverse applications in environmental fields and biomedical areas, such as tissue engineering, drug delivery systems, and biosensors.

## 4. Materials and Methods

### 4.1. Materials

Microcrystalline cellulose, titanium oxide (TiO_2_), iron (III) oxide (Fe_2_O_3_), chitosan (low molecular weight), κ-carrageenan, methyl orange (MO), and 1-ethyl-3-methylimidazolium acetate ([Emim][Ac]) were purchased from Sigma-Aldrich (St. Louis, MO, USA). Span 80, Congo red (CR), methylene blue (MB), crystal violet (CV), rhodamine B (RB), ethanol, isopropanol, hexane, and high-performance liquid chromatography (HPLC) grade water were purchased from Samchun Pure Chemical Co., Ltd. (Gyeonggi-do, Republic of Korea). Multi-walled carbon nanotubes (CMP-310F) were obtained from Hanwha Nanotech Co., Ltd. Incheon, Republic of Korea). All other chemicals used in this study were of analytical grade and used without further purification.

### 4.2. Preparation of Biopolymer/TiO_2_ Hydrogel Beads

**Figure 6:**
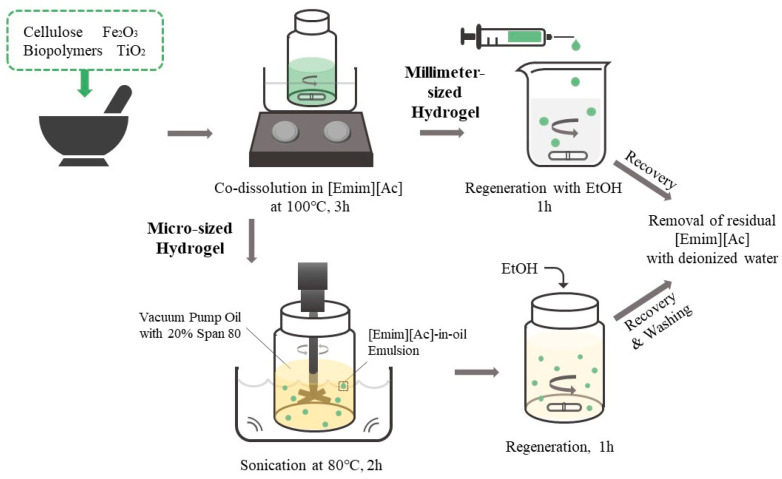
Schematic representation of the preparation process of biopolymer/TiO_2_ composite hydrogel beads.

Millimeter-sized biopolymer/TiO_2_ hydrogel beads were prepared using the sol–gel transition method with [Emim][Ac] as the solvent (Figure 6). Cellulose (7% *w*/*v*), TiO_2_ (0.5% *w*/*v*), and 0.5% (*w*/*v*)-modifying additives (CNT, chitosan, or κ-carrageenan) were added to [Emim][Ac] and mixed in a mortar. The blended solutions were then incubated at 100 °C, with stirring for 3 h. For the fabrication of millimeter-sized hydrogel beads, 1 mL of the prepared mixture solution was added dropwise into ethanol using a 1 mL syringe with a 26-gauge needle and syringe pump (LSP01-2A; Longer Pump, Baoding city, China) at a rate of 50 μL/min, with stirring. The hydrogel beads were cured in ethanol for 1 h, washed with deionized water (DW), and then washed again with DW until the removal of [Emim][Ac] was confirmed by measuring the optical density of the washing solution at 211 nm [15].

### 4.3. Preparation of Biopolymer/TiO_2_ Hydrogel Microbeads

Biopolymer/TiO_2_ hydrogel microbeads were prepared via the sol–gel transition method using an [Emim][Ac]-in-oil emulsion (Figure 6) [34]. Cellulose (7% *w*/*v*), TiO_2_ (0.5%, 2%, 3.5%, 5%, or 7% *w*/*v*), Fe_2_O_3_ magnetic particles (0%, 0.1%, 0.5%, 0.75%, or 1% *w*/*v*), and 0.5% (*w*/*v*) modifying additives (CNT, chitosan, or carrageenan) were added to [Emim][Ac] and mixed in a mortar. The blended solutions were then incubated at 100 °C, with stirring for 3 h. To fabricate the microbeads, 3 mL of the prepared solution was added to 30 mL of vacuum pump oil containing 20% (*v*/*v*) Span 80, with mechanical stirring at 80 °C, followed by sonication in a sonic bath for 2 h. The resulting emulsion was slowly cooled to room temperature. Ethanol was added to the [Emim][Ac]-in-oil emulsion under stirring, and the mixture was stirred for an additional 1 h to regenerate cellulose and biopolymers. The resulting hydrogel microbeads were harvested by centrifugation, and the liquid phase was removed. The collected hydrogel microbeads were washed in the following order: *n*-hexane, isopropanol, aqueous ethanol, and water to remove vacuum oil, surfactant, and [Emim][Ac]. The prepared hydrogel microbeads were stored in HPLC-grade water until further use.

### 4.4. Characterization of Biopolymer/TiO_2_ Hydrogel Beads

The dry weight of the biopolymer/TiO_2_ hydrogel beads was measured with 30 beads of millimeter size or 2 mL of dispersed microbead solution after drying at 60 °C. The mean diameter of the hydrogel microbeads was measured using a particle size analyzer (Mastersizer 2000; Malvern, UK). To investigate the surface of the biopolymer/TiO_2_ composite hydrogel beads, they were frozen overnight at −70 °C and then dried at −80 °C under vacuum for 24 h. All freeze-dried samples were sputter-coated with gold before surface observation under a scanning electron microscope (Carl Zeiss, Oberkochen, Germany). The XRD patterns of the hydrogel beads were analyzed after grinding the freeze-dried samples using a homogenizer. The samples were scanned using a D8 Advance Diffractometer (Bruker, Madison, WI, USA) at 40 kV and 40 mA. The 2θ angle was scanned from 10° to 40° at a width of 0.1°.

### 4.5. Adsorption and Photodegradation of Dyes

To determine the adsorption capacity and photodegradation activity of the biopolymer/TiO_2_ composite hydrogel beads, the hydrogel beads (5 mg or 10 mg as dry weight) were immersed in 4 mL of dye solutions with shaking at 80 rpm under dark conditions at 25 °C until equilibrium was reached. After the adsorption process reached equilibrium, the hydrogel beads were exposed to UV light (20 W BL lamp; ALIM, Republic of Korea) for 6 h. Aliquots were harvested periodically, diluted with water, and centrifuged to obtain the supernatants. The dye concentrations in the sample solutions were calculated by measuring absorbance using a spectrophotometer. The extinction coefficients of MB, MO, CR, CV, and RB were 74,000 (664 nm), 21,600 (464 nm), 270,000 (499 nm), 87,000 (590 nm), and 106,000 (555 nm) cm^−1^ M^−1^, respectively. All experiments were conducted three times, and the results were averaged.

The adsorption capacity of the biopolymer/TiO_2_ hydrogel beads (q_e_, mg/g) was calculated using the following equation:

(1) $$q_{e}=\frac{\left(C_{0}-C_{e}\right)}{m}\times V,$$

where C_0_ is the initial dye concentration (mg/L), C_e_ is the equilibrium concentration of the dye solution (mg/L), m is the weight of the beads (g), and V is the volume of the adsorption solution (L).

The photocatalytic activities of the biopolymer/TiO_2_ hydrogel beads (k, min^−1^) were determined using the following equation, fitted with the change in dye concentration (Figure S4):

(2) $$\ln\left(\frac{C_{0}}{C_{t}}\right)=kt,$$

where C_0_ is the initial concentration of the dye (mg/L), C_t_ is the remaining concentration of the dye solution (mg/L), k is the reaction rate constant (min^−1^), and t is the time (min).

## Figures and Tables

**Table 1:** Adsorption and photodegradation capacity of biopolymer/TiO_2_ hydrogel beads in aqueous methylene blue (MB) solution.

Biopolymers	Adsorption	Photodegradation	Dye Removal (%) ^a^
	q_e_ (mg/g) ^a^	k (×10^−3^/min) ^a^	
Cellulose	1.40 ± 0.06	1.17 ± 0.06	40.1
Cellulose/CNT	6.79 ± 0.04	1.22 ± 0.13	41.6
Cellulose/Chitosan	0.17 ± 0.07	1.05 ± 0.04	19.6
Cellulose/Carrageenan	9.74 ± 0.06	0.98 ± 0.05	67.4

^a^ Reaction conditions: 10 mg beads, 50 mg/L MB solution, and 16 h of adsorption in the dark, followed by 6 h of ultraviolet (UV) irradiation.

**Table 2:** Adsorption and photodegradation capacities of various biopolymer/TiO_2_ hydrogel beads in aqueous methyl orange (MO) solution.

Biopolymers	Adsorption	Photodegradation	Dye Removal (%) ^a^
	q_e_ (mg/g) ^a^	k (×10^−3^/min) ^a^	
Cellulose	0.20 ± 0.05	1.85 ± 0.09	54.8
Cellulose/CNT	1.63 ± 0.03	0.36 ± 0.11	38.6
Cellulose/Chitosan	0.96 ± 0.15	1.40 ± 0.10	58.0
Cellulose/Carrageenan	0.10 ± 0.05	1.70 ± 0.12	45.4

^a^ Reaction conditions: 5 mg beads, 10 mg/L MO solution, and 16 h of adsorption in the dark, followed by 6 h of UV irradiation.

**Table 3:** Effect of magnetic particle content in cellulose/TiO_2_ magnetic hydrogel microbeads on the adsorption and photodegradation capacity in aqueous methylene blue (MB) solution.

TiO_2_ (%):Fe_2_O_3_ (%)	Adsorption	Photodegradation	Dye Removal (%) ^a^
	q_e_ (mg/g) ^a^	k (×10^−3^/min) ^a^	
0.5:0	6.41 ± 0.11	3.23 ± 0.31	95.0
0.5:0.1	3.85 ± 0.56	1.30 ± 0.22	56.0
0.5:0.5	1.62 ± 0.15	1.36 ± 0.07	47.7
0.5:0.75	2.90 ± 0.18	0.67 ± 0.10	34.8
0.5:1.0	7.84 ± 0.14	0.33 ± 0.03	59.3

^a^ Reaction conditions: 10 mg beads, 50 mg/L MB solution, and 1 h of adsorption in the dark, followed by 6 h of UV irradiation.

**Table 4:** Effect of TiO_2_ content in cellulose/TiO_2_ magnetic hydrogel microbeads on the adsorption and photodegradation capacity in an aqueous methylene blue (MB) solution.

TiO_2_ (%):Fe_2_O_3_ (%)	Mean Diameter (μm)	Adsorption	Photodegradation		Dye Removal (%) ^a^
		q_e_ (mg/g) ^a^	k (×10^−3^/min) ^a^	k/mg TiO_2_ (×10^−3^/min/mg) ^a^	
0.5:0.5	22.3 ± 0.2	1.62 ± 0.15	1.36 ± 0.07	2.18 ± 0.11	47.7
2.0:0.5	25.9 ± 0.1	4.93 ± 0.21	1.49 ± 0.15	0.71 ± 0.07	66.8
3.5:0.5	34.6 ± 0.2	4.97 ± 0.42	2.18 ± 0.01	0.69 ± 0.00	77.4
5.0:0.5	37.7 ± 0.2	2.75 ± 0.29	1.88 ± 0.03	0.47 ± 0.01	59.5
7.0:0.5	52.6 ± 0.0	2.32 ± 0.20	1.23 ± 0.15	0.25 ± 0.03	48.1

^a^ Reaction conditions: 10 mg beads, 50 mg/L MB solution, and 1 h of adsorption in the dark, followed by 6 h of UV irradiation.

**Table 5:** Adsorption and photodegradation capacities of various biopolymer/TiO_2_ magnetic microbeads in aqueous methylene blue (MB) solution.

Biopolymers	Adsorption	Photodegradation	Dye Removal (%) ^a^
	q_e_ (mg/g) ^a^	k (10^−3^/min) ^a^	
Cellulose	1.62 ± 0.15	1.36 ± 0.07	47.7
Cellulose/CNT	10.34 ± 0.09	0.68 ± 0.04	65.4
Cellulose/Chitosan	1.51 ± 0.13	0.26 ± 0.17	32.0
Cellulose/Carrageenan	9.46 ± 0.06	0.53 ± 0.09	74.8

^a^ Reaction conditions: 10 mg beads, 50 mg/L MB solution, and 1 h of adsorption in the dark, followed by 6 h of UV irradiation.

**Table 6:** Adsorption and photodegradation capacities of various biopolymer/TiO_2_ magnetic microbeads in aqueous methyl orange (MO) solution.

Biopolymers	Adsorption	Photodegradation	Dye Removal (%) ^a^
	q_e_ (mg/g) ^a^	k (10^−3^/min) ^a^	
Cellulose	0.57 ± 0.15	1.28 ± 0.07	42.6
Cellulose/CNT	3.87 ± 0.12	0.62 ± 0.10	30.2
Cellulose/Chitosan	2.55 ± 0.35	1.58 ± 0.05	57.3
Cellulose/Carrageenan	0.10 ± 0.05	1.52 ± 0.10	48.3

^a^ Reaction conditions: 5 mg beads, 10 mg/L MO solution, and 1 h of adsorption in the dark, followed by 6 h of UV irradiation.

**Table 7:** Dye removal by various forms of cellulose/TiO_2_-based photocatalytic hydrogels.

Catalysts	Form	Dye (Concentration)	Time (h)	Dye Removal (%)	Ref.
Cellulose/TiO_2_	Microbeads	MB (10 mg/L)	6	95	this study
Cellulose/TiO_2_	Microbeads	MO (10 mg/L)	6	83	this study
Cellulose/TiO_2_	Microbeads	RB (10 mg/L)	6	85	this study
Cellulose/carrageenan/TiO_2_	Hydrogel film	MB (60 mg/L)	5	83	[26]
Cellulose/N-doped TiO_2_	Hydrogel film	MB (40 mg/L)	6	96	[27]
Cellulose/GO/TiO_2_	Hydrogel film	MB (10 mg/L)	2	93	[28]
Cellulose/TiO_2_/Fe_3_O_4_	Macrospheres	RB (12 mg/L)	1	24	[29]
Cellulose/TiO_2_	Monolith	MB (12 mg/L)	0.7	99	[30]
Cellulose/CMC/TiO_2_/Fe_3_O_4_	Monolith	MB (20 mg/L)	1	98	[31]
Cellulose/Cu_2_O/TiO_2_/rGO	Monolith	MO (20 mg/L)	2	85	[32]

## Data Availability

Not applicable.

## Supplementary Materials

The following supporting information can be downloaded from https://www.mdpi.com/article/10.3390/gels9080630/s1, Figure S1: SEM images of freeze-dried biopolymers/TiO_2_ beads. (a,b): cellulose; (c,d): cellulose/TiO_2_; (e,f): cellulose/chitosan; (g,h): cellulose/chitosan/TiO_2_; (i,j): cellulose/carrageenan; (k,l): cellulose/carrageenan/TiO_2_. Figure S2: X-ray diffraction patterns of (a) cellulose beads, (b) TiO_2_, and (c) cellulose/TiO_2_ beads. The contents of cellulose and TiO_2_ in the hydrogel bead-forming solutions were 7% and 0.5%, respectively. Figure S3: Recovery of cellulose/TiO_2_/Fe_2_O_3_ hydrogel microbeads using a neodymium magnet. Figure S4: Plotting of “ln (C_0_/C_t_) vs. t” to determine the photodegradation activity of cellulose/TiO_2_ millimeter-sized hydrogel beads (●) and cellulose/TiO_2_ hydrogel microbeads (○).
