# Supplementary material for: Development of Blended Biopolymer-Based Photocatalytic Hydrogel Beads for Adsorption and Photodegradation of Dyes
Source: Gels. 2023 Aug 5;9(8):630. doi: 10.3390/gels9080630 (PMC10454056; doi:10.3390/gels9080630)
Supplement: Supplementary file 1 [file gels-09-00630-s001.zip › gels-2528160-supplementary.pdf]

*Supplementary Material*

# Development of blended biopolymer-based photocatalytic hydrogel beads for adsorption and photodegradation of dyes

Seung Hyeon Weon[a,†], Jiwoo Han[a,†], Yong-Keun Choi[a,b], Saerom Park[a,b,*] and Sang Hyun Lee[a,*]

[a] Department of Biological Engineering, Konkuk University, Seoul 05029, Republic of Korea

[b] R&D Team, ChoiLab Inc., Seoul 01811, Republic of Korea

[†] These authors contributed equally to this work.

***Corresponding authors:**

Saerom Park

Tel.: +82-10-4038-4656

E-mail: angel4y@naver.com

Sang Hyun Lee

Tel.: +82-2-2049-6269

E-mail: sanghlee@konkuk.ac.kr

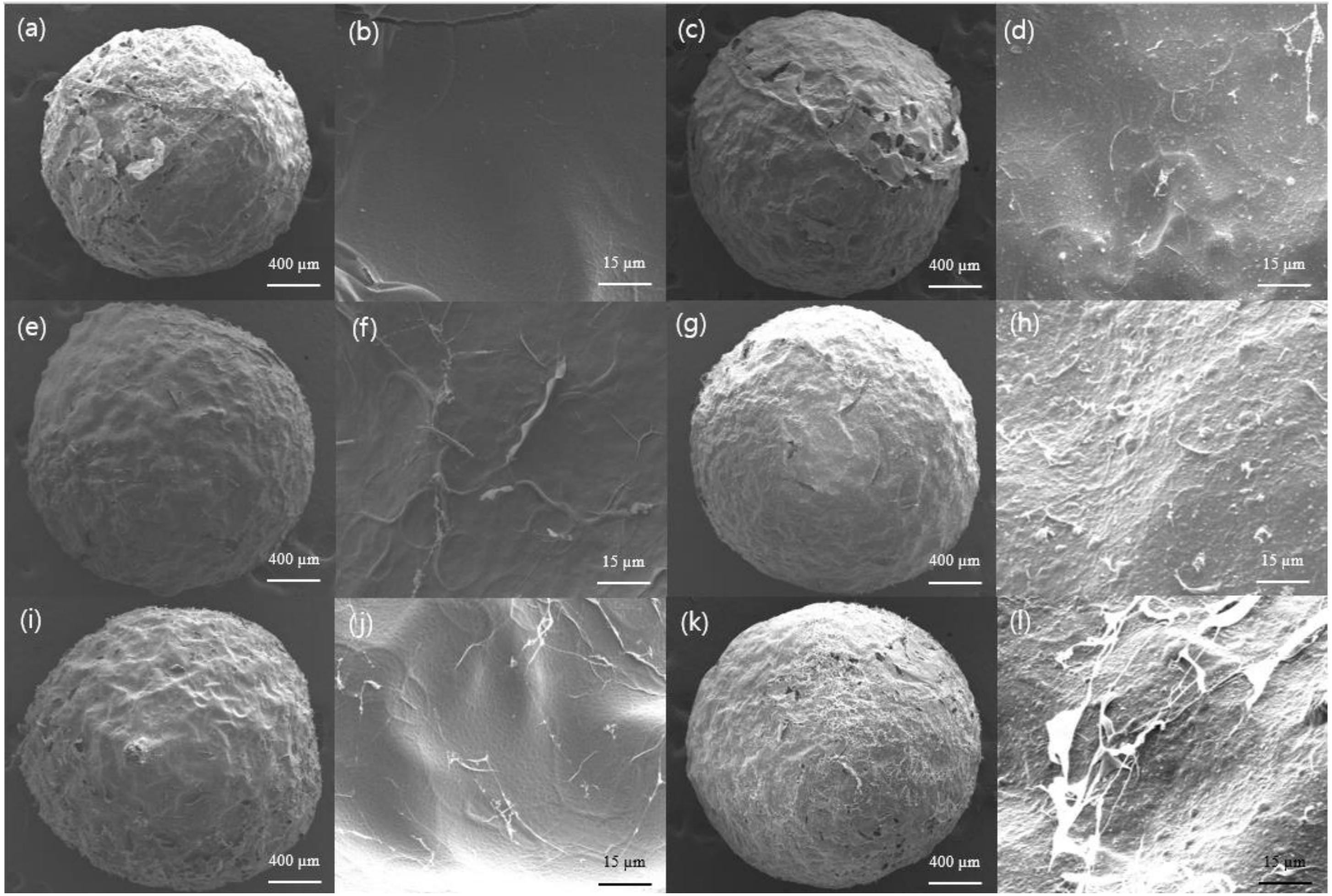


Figure S1. SEM images of freeze-dried biopolymers/$TiO_2$ beads. (a, b): cellulose; (c, d): cellulose/$TiO_2$; (e, f): cellulose/chitosan; (g, h): cellulose/chitosan/$TiO_2$; (i, j): cellulose/carrageenan; (k, l): cellulose/carrageenan/$TiO_2$.

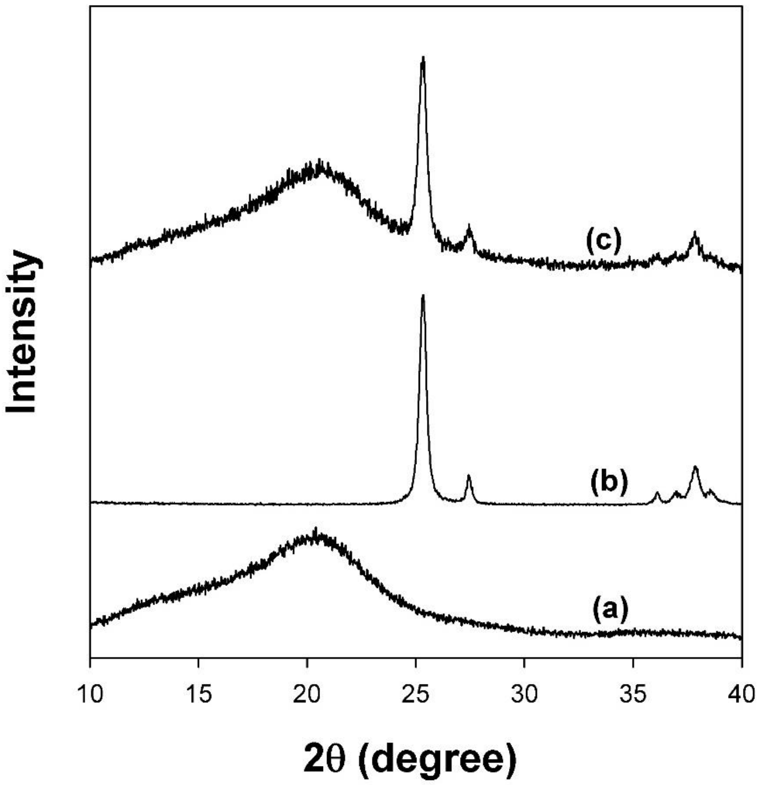


Figure S2. X-ray diffraction patterns of (a) cellulose beads, (b) $TiO_2$, and (c) cellulose/$TiO_2$ beads. The contents of cellulose and $TiO_2$ in the hydrogel beads forming solutions were 7% (w/v) and 0.5% (w/v), respectively.

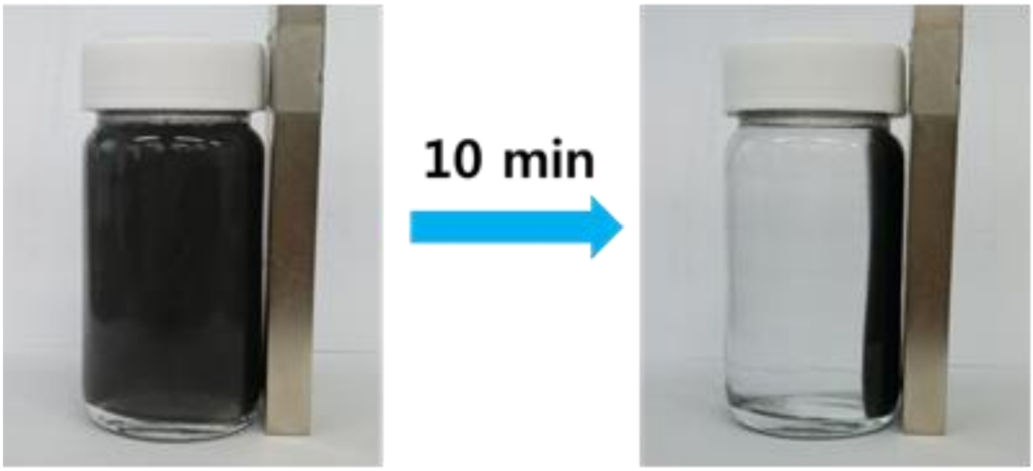


Figure S3. Recovery of cellulose/$TiO_2$/$Fe_2O_3$ hydrogel microbeads using a neodymium magnet.

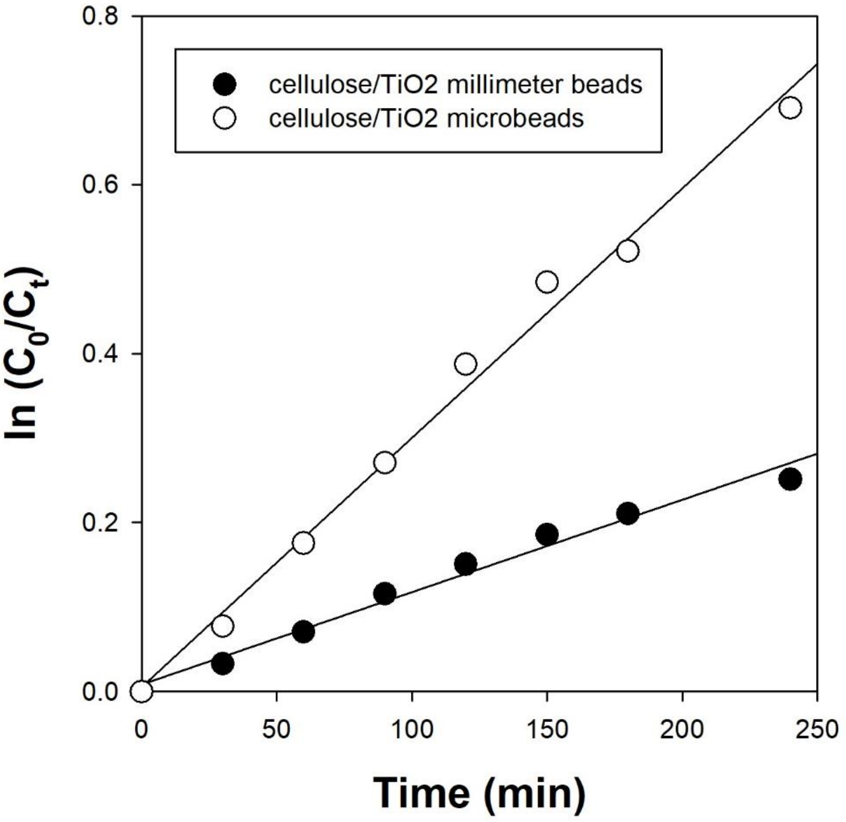


Figure S4. Plotting of “ln ($C_0/C_t$) vs. t” to determine the photodegradation activity of cellulose/$TiO_2$ millimeter-sized hydrogel beads (●) and cellulose/$TiO_2$ hydrogel microbeads (○).
